# Axillary vein as an alternative venous access site for VV-ECMO cannulation: a case report

**DOI:** 10.1186/s13019-024-02600-6

**Published:** 2024-03-13

**Authors:** Tao Pan, Xiaoyang Zhou, Jianneng Pan, Bixin Chen, Chang Xu, Zhaojun Xu, Pingping Dong, Tingting Yu

**Affiliations:** 1 Department of Intensive Care Medicine, Ningbo No.2 Hospital, Ningbo, 315000 Zhejiang China; 2 Baihe Street Community Health Services of Yinzhou District, Ningbo, 315000 Zhejiang China; 3 Department of Ophthalmology, Ningbo No.2 Hospital, Ningbo, 315000 Zhejiang China

**Keywords:** Venovenous extracorporeal membrane oxygenation, Vein thrombosis, Return cannula, Ultrasound-guided

## Abstract

**Background:**

Ultrasound-guided percutaneous axillary vein cannulation can reduce cannulation failure and mechanical complications, is as safe and effective as internal jugular vein cannulation, and is superior to subclavian vein cannulation using landmark technique. As far, reports of venovenous extracorporeal membrane oxygenation (VV-ECMO) with percutaneous axillary vein cannulation are rare.

**Case presentation:**

A 64-year-old man presenting with dyspnea and chest tightness after aspirating sewage was admitted to the emergency department. Computed tomography (CT) showed diffuse exudation of both lungs and arterial blood gas analysis showed an oxygenation index of 86. He was diagnosed with aspiration pneumonia-induced acute respiratory distress syndrome (ARDS) and intubated for deteriorated oxygenation. Despite the combination therapy of protective mechanical ventilation and prone position, the patient’s oxygenation deteriorated further, accompanied with multiple organ dysfunction syndrome, which indicated the requirement of support with VV-ECMO. However, vascular ultrasound detected multiple thrombus within bilateral internal jugular veins. As an alternative, right axillary vein was chosen as the access site of return cannula. Subsequently, femoral-axillary VV-ECMO was successfully implemented under the ultrasound guidance, and the patient’s oxygenation was significantly improved. Unfortunately, the patient died of hyperkalemia-induced ventricular fibrillation after 36 h of VV-ECMO running. Despite the poor prognosis, the blood flow during ECMO run was stable, and we observed no bleeding complication, vascular injury, or venous return disorder.

**Conclusions:**

Axillary vein is a feasible alternative access site of return cannula for VV-ECMO if internal jugular vein access were unavailable.

## Introduction

The purpose of venovenous extracorporeal membrane oxygenation (VV-ECMO) for acute respiratory distress syndrome (ARDS) is to provide extracorporeal gas exchange, while a protective ventilation strategy allows for pulmonary rest and recovery [1]. The classic cannulation strategy is right femoral vein–right internal jugular venous cannulation [2–5]. In clinical practice, there are other cannulation strategies, such as femoral vein–femoral venous cannulation, internal jugular vein double-lumen cannulation, and subclavian vein double-lumen cannulation, according to the patient’s condition, the difficulty of cannulation, and the experience of the ECMO team [6–10]. Femoral venous cannulation is associated with higher rates of infection, vascular and bleeding complications, and patient mobility limitations [11–13]. Although the internal jugular vein is superior to femoral vein access relative to avoid infection, this gives the patient limited head movement, which leads to increased bleeding complications and catheter migration. Subclavian vein cannulation brings the risk of complications such as pneumothorax and arterial perforation [14]. Percutaneous axillary central venous cannulation can reduce cannulation failure and mechanical complications, is as safe and effective as internal jugular vein cannulation, and is superior to subclavian vein cannulation using landmark technique [14–17]. As far, axillary artery access to VA-ECMO has been extensively described in the literature and has shown good results [18, 19], while percutaneous axillary vein cannulation for VV-ECMO has been rarely reported.

Here, we present a case of severe ARDS complicated with bilateral internal jugular vein thrombosis who were implemented with femoral–axillary VV-ECMO under ultrasound-guided percutaneous axillary vein cannulation, which alleviated the severe hypoxemia in the patient.

## Case report

**Fig. 1:**
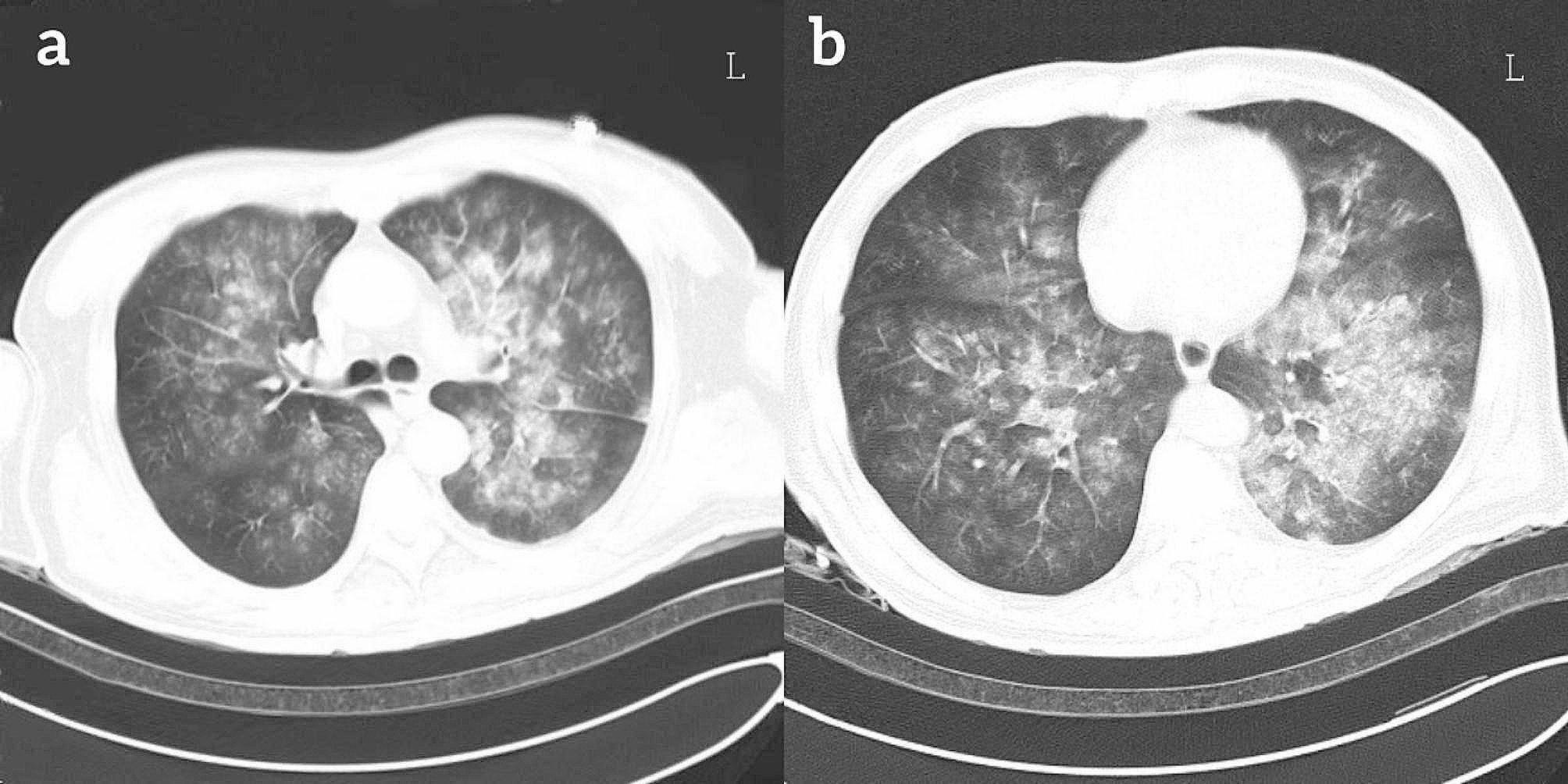
Chest computed tomography (CT) shows diffuse exudation of both lungs (**a**, **b**)

The patient was a 64-year-old male laborer. He came to the emergency department of our hospital and presented with dyspnea and chest tightness due to inhaling black sewage after falling into an elevator shaft. Computed tomography (CT) showed diffuse exudation of both lungs (Fig. 1) and no craniocerebral hemorrhage. Tracheal intubation and mechanical ventilation were administered in the emergency room. Arterial blood gas analysis showed an oxygenation index of 86. The patient was diagnosed with aspiration pneumonia-induced ARDS and then transferred to the intensive care unit (ICU) for treatment.

The patient was in a sedated and analgesic state at admission to the ICU. The temperature was 36.4℃, heart rate was 115 beats per minute, and blood pressure was 132/98mmHg. Heavy moist rales could be heard in both lungs. The heart rhythm is regular, with no pathological murmurs detected, and no lower limb edema observed. Arterial blood gas test showed pH of 7.32, lactate level of 2.4 mmol/L, partial pressure of oxygen 69mmHg, and oxygen saturation of 93%. The white blood cell count was 1.9 × 10^9/L, with an absolute neutrophil count of 0.9 × 10^9/L. Liver and kidney function, as well as coagulation function, were normal. No abnormalities were noted on the echocardiogram, and no inotropic support were being used. Bedside fiberoptic bronchoscopy revealed a moderate amount of gray, frothy sputum in the airway. Symptomatic and supportive treatment were given, including a protective mechanical ventilation, prone-position ventilation, fiberoptic bronchoscopy, lung recruitment, fluid management, anticoagulation, and anti-infection.

On the 6th day after admission to the ICU, the patient’s body temperature and inflammatory indices increased, and his oxygenation deteriorated. CT showed that the exudation of bilateral lungs had progressed more than before, indicating that the pulmonary infection was aggravated. His antibiotics were adjusted. However, the next day, the patient’s oxygenation continued to deteriorate, urine output was significantly reduced, and multiple organ dysfunction syndrome developed. The arterial blood gas test revealed pH of 7.15, lactate level of 7.1 mmol/L, partial pressure of oxygen of 70 mmHg, and oxygen saturation of 94% while the patient was on invasive mechanical ventilation with a fraction of inspired oxygen of 100%. The oxygenation index decreased to 70. Echocardiography was performed to assess cardiac function, which was found to be adequate. The ICU’s ECMO team quickly made the decision to start VV-ECMO.

**Fig. 2:**
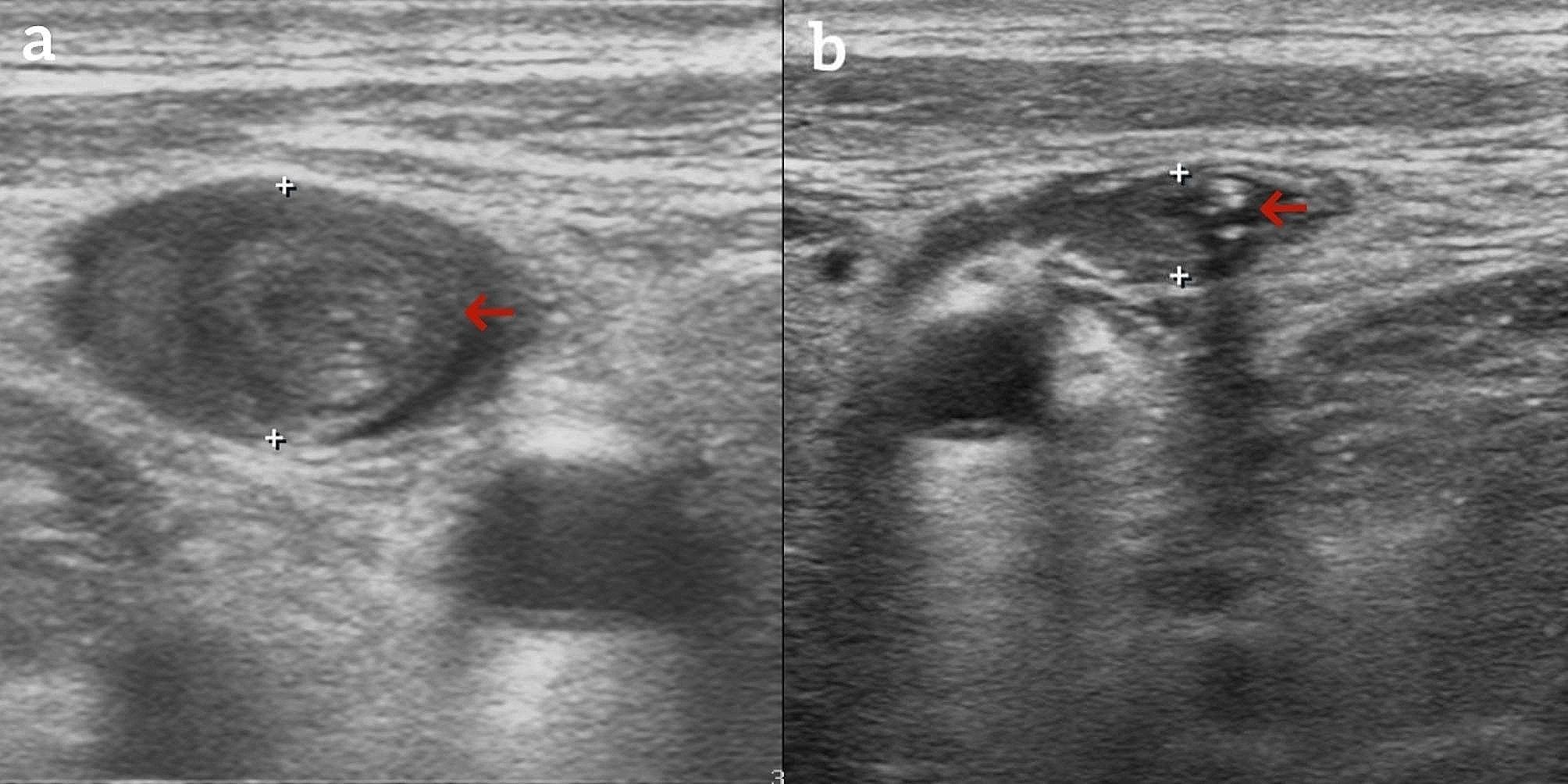
Internal jugular vein ultrasound shows thrombosis in bilateral internal jugular vein. a: thrombus (red arrow) in right internal jugular vein; b: thrombus (red arrow) in left internal jugular vein

**Fig. 3:**
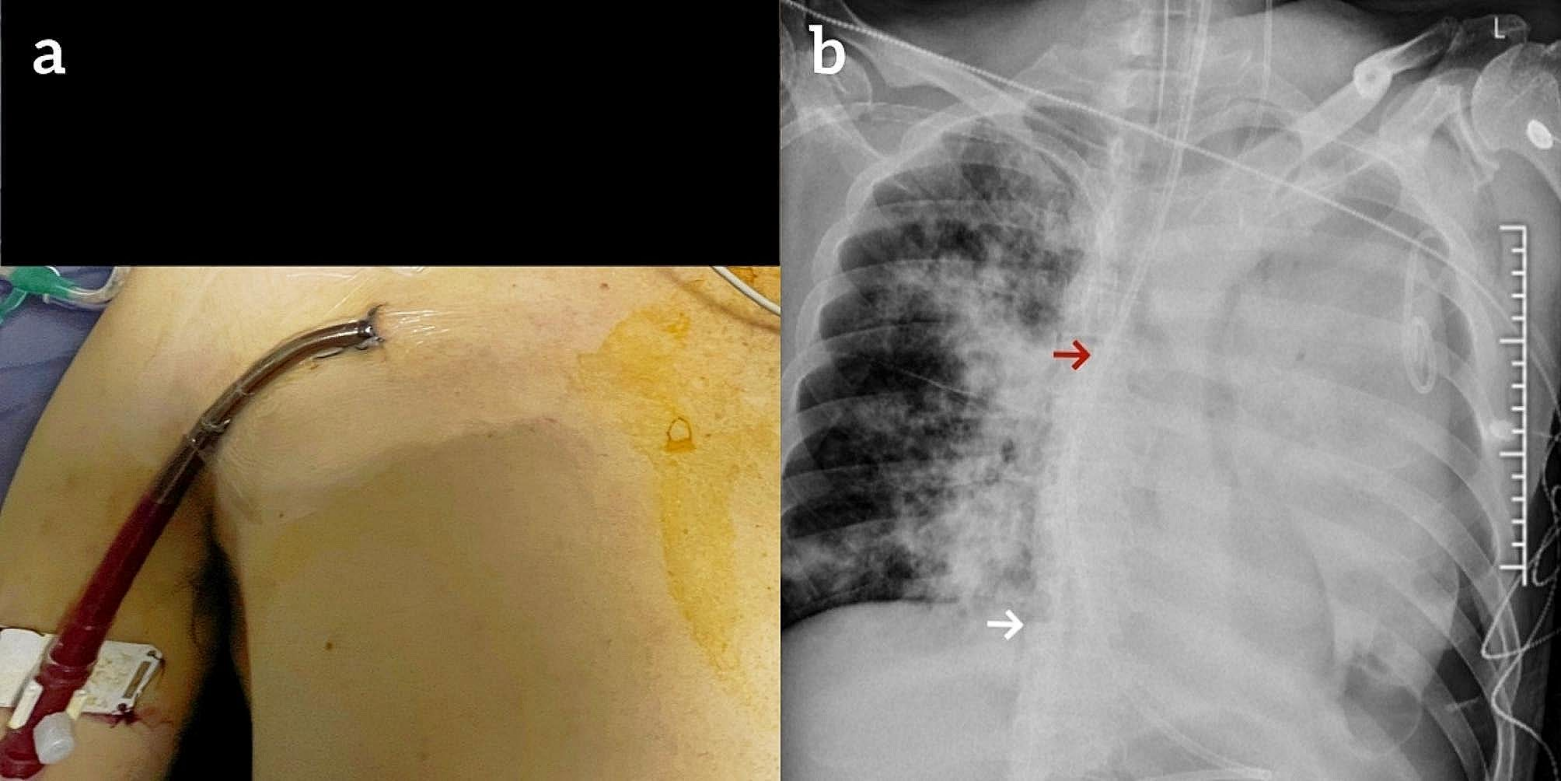
Axillary vein is punctured and inserted into the return cannula (**a**). Bedside chest X-ray shows the return cannula tip (red arrow) and the drainage cannula tip (white arrow) (**b**)

During the ultrasound evaluation before ECMO cannulation, the patient had bilateral internal jugular vein thrombosis (Fig. 2). No thrombus floating in the superior vena cava was observed. After discussion, we decided to perform VV-ECMO via femoral–axillary venous cannulation. A preoperative scanning was conducted to examine the axillary vein in the long axis and short-axis view. The long-axis, in-plane, real-time, ultrasound-guided approach was used in which the axillary vein and the entire needle were simultaneously visualized during cannulation. When introducing the needle, the progression of the needle into the vein was visualized in real time. Under the ultrasound guidance, we used Maquet HLS cannulae and percutaneous insertion kits, to perform the modified Seldinger technique for percutaneous cannulation. The proximal right femoral vein was punctured, and a 23-F drainage catheter (Maquet Cardiopulmonary AG, Rastatt, Germany) was run from the right femoral vein to the right atrium at a depth of 40 cm. Using the same technique, a 19-F return catheter (Maquet Cardiopulmonary AG, Rastatt, Germany) was inserted into the right axillary vein at a depth of 15 cm. Echocardiographic guidance was used for positioning of wire and cannula. No ventricular arrhythmias were observed during the placement of the wire and cannula. The location of the axillary vein cannula was confirmed by bedside chest X-ray (Fig. 3). VV-ECMO pump was started at 2700 rpm, a blood flow of 3.8 L/min, an oxygen flow of 3 L/min, and oxygen concentration of 100%.

After starting VV-ECMO, we initiated ultra-protective ventilation and the ventilator settings as follows: pressure controlled ventilation mode, with a fraction of inspired oxygen of 40%, plateau pressure of 15cmH2O, positive end expiratory pressure of 5cmH2O, respiratory rate of 10 breaths per minute, and an inspiratory to expiratory ratio of 1:1. Meanwhile, concurrent continuous renal replacement therapy was started, with the dialysis catheter connected to the ECMO circuit. Unfractionated heparin is used for systemic anticoagulation to prevent thrombotic events. After ECMO initiation, the arterial blood gas test showed pH of 7.37, lactate level of 4.6 mmol/L, partial pressure of oxygen of 72mmHg, and oxygen saturation of 97%. The oxygenation index improved to 180. Vascular ultrasound showed unobstructed blood flow, and no increase in CPK level. According to the results of sputum and blood tests, it was found that the patient was infected with multiple pathogens, including klebsiella pneumoniae, stenotrophomonas maltophilia, aeromonas veronii, aspergillus flavus, among others. We administered meropenem, tigecycline, sulfamethoxazole, and voriconazole for the anti-infection. Unfortunately, the pulmonary infection continued to advance and led to septic shock, multiple-organ dysfunction, including liver failure, renal failure, and respiratory failure. In the end, the patient died of hyperkalemic ventricular fibrillation after 36 h on VV-ECMO. During the VV-ECMO support period, ECMO blood flow was stable without any complications, such as bleeding, vascular injury, or obstruction of venous return.

## Discussion and conclusion

In this case report, we describe a patient with severe ARDS and bilateral internal jugular vein thrombosis who underwent successful femoral–axillary VV-ECMO with stable flow during ECMO support without any complications such as bleeding, vascular injury, or obstruction of venous return. Reports of percutaneous axillary vein cannulation for VV-ECMO are rare. Our report demonstrates the feasibility of VV-ECMO with axillary vein cannulation.

Studies have reported a high rate of recirculation for femoral–femoral VV-ECMO [5], which is associated with higher rates of infection, vascular and bleeding complications, and patient mobility limitations [11–13]. Although the jugular vein is superior to the femoral vein for avoiding infection, this gives the patient limited head movement, which can increase the risks of bleeding, catheter displacement, and dislocation. Subclavian vein cannulation has a low risk of infection, high patient comfort, and ease of care. However, due to the difficulty displaying the clavicle on ultrasound, subclavian vein cannulation is more difficult than internal jugular vein and femoral vein cannulation and may bring a risk of perforating arteries and pneumothorax [14]. The axillary vein runs outside the thorax and continues as the subclavian vein. The puncture sites of the axillary vein and distal subclavian vein are very similar, but the approaches and risks are different between the two techniques. Compared to subclavian vein cannulation, ultrasound-guided axillary vein cannulation offers better visualization. It carries a lower risk of arterial, pleural, and nerve damage. Additionally, possibility to apply pressure in case of arterial puncture. The average diameter (standard deviation) of the axillary vein is 12 (3) mm on the right and 11 (2) mm on the left [20]. Therefore, the return cannulas within 24-F can be considered for ECMO cannulation.

The rare use of axillary vein cannulation in VV-ECMO may be attributed to several reasons. Firstly, when the right internal jugular vein was unavailable, the left internal jugular vein, left femoral vein, and bilateral subclavian veins will be preferred. Secondly, some patients’ axillary vein is too narrow for cannulation. Additionally, some ECMO centers have not adopted percutaneous axillary vein cannulation technology. Moreover, compared with femoral vein and internal jugular vein cannulation, axillary vein cannulation is difficult and time-consuming. Lastly, there is not enough experience or research on axillary vein cannulation in VV-ECMO cannulation strategies. Even so, we believe that ultrasound-guided percutaneous axillary vein cannulation has potential application value in ECMO. Firstly, for awake ECMO patients, it may has higher comfort and fewer activity limitations, which is helpful for recovery. Moreover, for tracheotomy patients, in theory, staying away from the stoma may reduce the contamination of the cannula. Finally, for patients with burns, trauma, vascular thrombosis, and inferior vena cava filter implantation, when other deep veins cannot be accessed, the axillary vein may be the last but only alternative. Of course, we also need to be aware of the technical pitfalls of ultrasound-guided axillary vein puncture, firstly, the operator requires time to learn and train ultrasound-guided puncture techniques to minimize complications. In addition, subcutaneous emphysema or obesity in the patient can result in poor ultrasound imaging. Furthermore, the axillary vein lacks soft tissue attachments, so in cases of low blood volume, collapse may be observed during inspiration, leading to puncture failure. Lastly, deeper and narrower veins may make it difficult to pass wire and cannula [21].

In this patient, vascular ultrasound evaluation revealed bilateral internal jugular vein thrombosis, a contraindication to cannulation. Since our ECMO center has not introduced a dual-lumen cannula, we eschewed a strategy of double-lumen venous cannulation. To minimize the number of repeated punctures and potential vascular damage, we utilized the long-axis, in-plane, real-time, ultrasound-guided approach to the axillary vein. Based on the patient’s axillary vein diameter of 1 cm, we chose a 19-F return cannula whose diameter is less than two-thirds of the vessel diameter to minimize any obstruction in venous return. After cannula placement, a vascular ultrasound examination was performed to ensure unobstructed blood flow. After ECMO initiation, the blood flow was set at 3.8 L/min, considering the patient’s weight of 65 kg, to meet the target of 60 ml/kg/min. Blood gas test showed oxygen saturation of 97%, hemoglobin was 100 g/L, lactate decreased to 4.6 mmol/L. We believe that the oxygen delivery meets the patient’s needs.

The patient underwent difficult central venous catheterization of bilateral internal jugular veins with multiple punctures and compression for hemostasis, the day before VV-ECMO support. After the placement of the catheter, a follow-up bedside chest X-ray showed left pneumothorax and a chest tube was inserted for drainage. We believe that thrombosis and pneumothorax are related to this procedure. The patient developed severe pneumonia and ARDS due to inhalation of sewage. The severity of lung lesions was not consistent between the left and right lungs (Fig. 1). Additionally, there was also a left-sided pneumothorax. Therefore, there was a strongly difference in the appearance of the lungs on the chest X-ray between the left and right sides (Fig. 3b). The patient had already experienced a significant decrease in urine output before being placed on VV-ECMO. After the initiation of ECMO, we performed simultaneous hemodialysis filtration to reduce potassium level. Unfortunately, the patient developed septic shock and died of hyperkalemic induced ventricular fibrillation after 36 h of VV-ECMO running. There were no signs of congestion or swelling in the patient’s right upper and lower limbs. Vascular ultrasound showed unobstructed blood flow, and no increase in CPK level. We believed that infection and severe hypoxemia were responsible for the acute renal failure and hyperkalemia, with septic shock as the aggravating reason. The patient received anticoagulation with low molecular weight heparin before ECMO initiation and systemic anticoagulation with unfractionated heparin during ECMO support. Regrettably, a computed tomography pulmonary angiogram could not be performed. However, ECG and echocardiography did not show typical signs of pulmonary embolism. Therefore, there was insufficient evidence to support that the death was due to pulmonary embolism.

Despite the poor prognosis, VV-ECMO alleviated the severe hypoxemia due to ARDS in this patient. During VV-ECMO treatment, the flow rate was stable, and no complications, such as bleeding, vascular injury, or obstruction of venous return, occurred. This case report conveys our clinical experience that the axillary vein is an approach worth considering in patients who are unsuitable for internal jugular vein cannulation and require VV-ECMO. It should be noted that this report includes only one case, which is a major limitation. In addition, more studies are required to address axillary venous access and the comfort or the recovery of awake VV-ECMO patients. Therefore, the results should be applied to clinical practice with caution.

In conclusion, the axillary vein can be used as an alternative access site for VV-ECMO cannulation when internal jugular vein cannulation cannot be performed.

## Data Availability

Data sharing not applicable to this article as no datasets were generated or analyzed during the current study.

## Abbreviations

VV-ECMO: Venovenous extracorporeal membrane oxygenation
ARDS: Acute respiratory distress syndrome
CT: Computed tomography
ICU: Intensive care unit
